# Disability-inclusive disaster risk reduction in schools: A confirmatory factor analysis

**DOI:** 10.4102/jamba.v16i1.1559

**Published:** 2024-07-11

**Authors:** Nurul H. Rofiah, Norimune Kawai, Elli N. Hayati

**Affiliations:** 1 The Institute for Diversity and Inclusion, Hiroshima University, Higashi-Hiroshima, Japan; 2 Department of Psychology, Graduate for Psychology, Universitas Ahmad Dahlan, Yogyakarta, Indonesia

**Keywords:** disaster risk reduction, inclusive education, children with special needs, disabilities, hazard

## Abstract

This research examines the validity and reliability of the construct variables and indicators of the disability-inclusive disaster risk reduction (DiDRR) model in the school environment. It also aims to determine the contribution of aspects and indicators in measuring variables and confirm the hypothesised model’s suitability with empirical data. The explanatory research uses the purposive sampling method to collect questionnaire data from principals and teachers (*n* = 147) of primary schools in Yogyakarta Province, Indonesia. The results showed that the factors responsible for the formation of DiDRR in school include identifying and supporting children with special needs, accessibility, meaningful participation, non-discrimination, strategy and method, collaboration and networking. Although each factor has a significant causative relationship with the DiDRR variable, accessibility is the strongest forming factor. The modified model meets the goodness of fit criteria, thereby leading to a DiDRR model for elementary schools in inclusive settings. The research only considered principals and teachers in Yogyakarta province. Therefore, further research needs to be carried out in other provinces and widen its potential generalisation of findings.

**Contribution:**

The research identified and examined the shaping factors of DiDRR in schools from the perspective of teachers and principals. Testing of the construct in the DiDRR model is intended to give more valid information about the concepts. It also acts as a guide when implementing it in schools to reduce risk and achieve broader targets for children with special needs and disabilities.

## Introduction

During disasters, persons with disabilities (PWDs) have the right to self-defense and survival. The state is required to ensure PWDs’ protection and well-being in perilous situations, such as armed conflict, humanitarian emergencies and natural hazard, in accordance with Article 11 of the Convention on the Rights of Persons with Disabilities (United Nations 2006). Persons with disabilities have the same rights to risk reduction and management measures as any other citizen and have the right to participate equally in the design and implementation of disaster risk reduction (DRR) policies and practices (Calgaro, Villeneuve & Roberts 2020). However, PWDs are less involved in emergency preparedness and response efforts, and therefore existing disaster preparedness programmes are not sensitive to their needs (Handicap International 2014).

In Indonesia, the National Disaster Risk Management Regulation number 14 of 2014 addresses the handling, protection and participation of PWDs, thereby establishing a policy that regulates their right to protection during disasters. According to the regulation, people with disabilities must receive priority in emergency management operations. However, in practice, this regulation is not always aligned with the disaster management efforts implemented in the field (Fatdri & Alhadi 2020; Winarno, Rusmiyati & Probosiwi 2021).

Schools are a gathering place for vulnerable groups, particularly children, including those with special needs and disabilities (Boon et al. 2012; Miresmaeeli et al. 2021; Rofiah, Kawai & Hayati 2021). Ronoh, Gaillard and Marlowe (2015) found that initiatives for disaster preparedness tend to ignore children with disabilities. Hence, these children often have difficulty accessing resources during a disaster. Several studies found that students with disabilities deserve active participation in DRR education (Edmonds 2017; Nikolaraizi et al. 2021; Ronoh 2017; Ronoh et al. 2015). A multi-case study conducted at several schools in New Zealand’s Auckland region reported that schools provided an accessible, safe and inclusive learning environment for children with diverse abilities (Ronoh, Gaillard & Marlowe 2017). However, these schools still lacked inclusiveness in their DRR planning and approach (Boon et al. 2011). In schools, disability-inclusive DRR should acknowledge diversity and ensure that all marginalised and excluded individuals, such as disabled children, participate and contribute as stakeholders in the process (Ronoh et al. 2017; Stough et al. 2020).

Because education plays a strategic role, disaster risk reduction initiatives are integrated into school curricula and extracurricular activities. Several studies have been conducted on the tools used for making safe and resilient schools. For instance, Widowati, Istiono and Husodo (2021) developed a disaster preparedness and safety school model to increase resilience against multihazard threats from natural hazard, dangers of inadequate security systems and violence against children. The formation factors of the model include school commitment, formal education curriculum, information disclosure, facilities and infrastructure, readiness, supervisory system, empowering the role of institutions, and the members’ ability. Disaster preparedness and safety school is significantly correlated with each of the factors. Dwiningrum (2017) conducted research to develop school resilience for disaster mitigation in Indonesia. The model appropriately explained risk factor minimisation and environmental resilience development. The variables explored important aspects, including (1) increasing bonding, (2) setting clear and consistent boundaries, (3) teaching life skills, (4) providing attention and support, (5) setting and communicating high expectations, and (6) providing meaningful participation opportunities.

However, from the preceding descriptions, the disaster preparedness and safety school model and the School resilience model in Indonesia do not include the disability-inclusive disaster risk reduction (DiDRR) in schools yet.

The DiDRR is concerned with ensuring that PWDs have equal access to emergency preparedness information, participate in their community’s emergency preparedness programmes and are valuable stakeholders in other phases (prevention, preparedness, response and recovery) of local community disaster risk management (Villeneuve et al. 2021). Therefore, this study examines the validity and reliability of construct variables and indicators of DiDRR model in schools. It also aims to determine the contribution of aspects and indicators in measuring variables and confirm the hypothesised model’s suitability using empirical data. Testing of the construct in the DiDRR model is intended to give more valid information about the concepts. It also acts as a guide when implementing it in schools to reduce risk and achieve broader targets for children with special needs and disabilities.

## Literature review

### Disability and disaster in Indonesia

Indonesia is prone to earthquakes because of its location at the intersection of three tectonic plates: the Indo-Australian, Eurasian and Pacific Plates (National Disaster Management Authority [NDMA] 2018). With 127 active volcanoes, the nation has the most active volcanoes in the world. Some of these volcanoes have erupted with the most force in human history (NDMA 2018). Indonesia also has the world’s longest coastline and, consequently, high exposure to tsunami risk along the coast (NDMA 2018). In addition, Indonesia’s tropical climate makes it susceptible to flooding, landslides, extreme weather, drought and forest fire, as well as beach erosion and extreme waves in numerous locations (NDMA 2017). The country’s rapid development and population growth also place it at high risk of industrial accidents and disease outbreaks (Ministry of Education and Culture 2017).

In Indonesia, this right of PWDs to be disaster safe is regulated by Law Number 8 of 2016 Article 29, consisting of (1) obtaining easily accessible disaster information and knowledge on risk reduction, (2) prioritising the rescue process and evacuation in a disaster situation, (3) providing easy and accessible evacuation processes, and (4) prioritising facilities easily accessible at evacuation sites. Meanwhile, from Indonesia Law Number 24 of 2007 concerning disaster management, PWDs are still seen as objects of recipients of assistance and compensation (charity). Embedded in Law Number 8 of 2016 are two articles making reference to people with disabilities. The first, Article 55, talks about the vulnerable groups that should be rescued first in emergency and post-disaster situations. The second, Article 69, explains that both the federal government and the local governments are obligated to provide assistance in the form of compensation to victims who die or are disabled. Therefore, efforts to participate in disaster management and preparedness are not accommodated in the law.

### The concept of disability-inclusive disaster risk reduction

The UN Department of Economic and Social Affairs (UNDESA) held a public forum on 15 March 2015, called ‘Taking Action Towards a Disability-Inclusive Disaster Risk Reduction Framework and Its Implementation (Izutsu 2019)’. The Public Forum talked about and made suggestions for DiDRR as a contribution to the Sendai Framework for Disaster Risk Reduction. The forum likewise did a stocktaking, survey, and evaluation of the viability of existing debacle risk decrease strategies and projects, as well as an assessment of the progress made, and lessons learned to advance DiDRR at the local, national, regional, and international levels (Izutsu 2019). It was also meant to strengthen and expand the networks of the people who took part so that they could work together more to make sure that issues related to disability were part of global development efforts (United Nations Office for Disaster Risk Reduction [UNISDR] 2014). Since then, varying degrees of progress have been made in mainstreaming disability in DRR, as well as in the implementation of DiDRR, which has led to the discovery of additional best practices and lessons (Izutsu 2019).

People with disabilities are included as valuable stakeholders in all phases of local community DRR, including early warning system prevention, preparedness, response, recovery and rehabilitation as DiDRR ensures that they have equal access to emergency preparedness information and programmes (Pertiwi et al. 2019; Villeneuve et al. 2021). Furthermore, it relies on effective cross-sectoral collaboration between emergency managers and community services, as well as disability support personnel working for people with disabilities. Families and allies must remove obstacles that prevent PWDs from participating in DRR activities. The critical parameters of DiDRR are initiatives, comprehensive accessibility, universal design, non-discrimination of facilities, collaboration and cooperation (Alburo-Canete & Pasicaran 2016; Fuhrer 2014; GFDRR 2017; Ronoh et al. 2017). This has led to the development of five key inclusion models by Arbeiter Samariter Bund (ASB), which consisted of identifying children with special needs, accessibility, participation, capacity building and priority protection (ASB Indonesia and the Philippines 2018).

In another study, Rofiah et al. (2021) discussed six key elements associated with the implementation of inclusive disaster mitigation education in Indonesian schools from the stakeholders’ perspective. These encompass (1) a strong commitment to initiating disaster risk reduction (DRR) education for all students, (2) adjustments to infrastructure and the learning environment to support children with special needs, (3) expanding the methods of DRR education, (4) empowering children and encouraging their meaningful participation, (5) raising awareness of school management and DRR strategies and (6) engaging a wide range of stakeholders in disaster mitigation education. The statement also emphasises that the critical elements are intimately linked to one another (Rofiah et al. 2021). To accommodate students with special needs and other students and guarantee their active participation, the infrastructure and learning environment must be modified. Through the initiative to identify the needs of children with disabilities, who will participate, and their capacities and difficulties, the appropriate data are also required to provide proper access. Expanding learning methods to increase meaningful participation is also related to accessibility. Stakeholder networks that work together can raise awareness of school management and DRR strategies.

Through a collaborative effort and inclusion mechanisms on the appropriate emergency management procedure to improve resilience, the ultimate objective of DiDRR is to facilitate equitable access for PWDs. The Sendai Framework for Disaster Risk Reduction emphasised the need for cross-sectoral collaboration to create opportunities for collaboration and incorporate disaster risk management into routine community activities (Quaill, Barker &West 2019).

### Identification of children with special needs and disability-inclusive disaster risk reduction

Proponents of disability inclusion believe that having accurate statistics is ‘the first step towards inclusion’ (Sloman & Margaretha 2018). Data on disability help to provide better knowledge about the affected persons, possible dangers and strategies used to support and accommodate them to ensure inclusion. Furthermore, it is critical to assess information on community hazards, infrastructure, services and the ability to manage identified risks in the context of disaster risk reduction (ASB Indonesia and the Philippines 2018). To this end, schools play a crucial role in gathering this vital data.

The data collected from schools involve identifying the diversity, abilities and strengths of children with special needs for diagnosis and treatment in line with their medical history (Ministry of Education and Culture 2020). This identification must be conducted carefully to avoid incorrect actions regarding the children’s physical and/or mental conditions, ensuring that the identification process leads to appropriate follow-up (Rofiah 2015). By carefully collecting and analysing this data, schools can contribute significantly to the broader effort of disaster risk reduction and inclusive education.

### Accessibility and disability-inclusive disaster risk reduction

Accessibility expands opportunities for people with or without disabilities to engage and contribute actively (Axelsson 2021; CBM 2018). It also makes it easy for all parties to access and utilise services according to their needs and capacities. Furthermore, it enables everyone to participate in disaster capacity-building activities and collectively contribute to comprehensive risk reduction. There are two types of accessibility, namely: non-physical and physical, both of which allow everyone to carry out their activities safely, efficiently, and independently without discrimination (Kruger et al. 2018). Non-physical accessibility allows everyone to easily enter, use, and exit a system, while physical accessibility is carried out within a building.

Inclusive education in schools provides integrated education services for children with special needs. It is also important because it provides accessibility for daily activities and easy evacuation during a disaster. Without inclusive education, there is a possibility of hampering the evacuation process. Ease of mobility relates to infrastructural development, such as the construction of ramps for wheelchair users, installation of guide lanes for the blind, pedestrian paths and accessible toilets (Margaretha 2020).

### Meaningful participation and disability-inclusive disaster risk reduction

Meaningful participation empowers all community groups by providing equal opportunities to make various decisions. It supports people who are barely involved in activities, discussions and decision-making to actively participate and provide opinions (Ton et al. 2019). This process gives everyone, with or without disabilities, an opportunity to participate in planning, implementation, monitoring, evaluation and making decisions on initiatives and services. Individuals with disabilities who are fully involved in both the delivery and planning of disaster-related services not only generate better disaster preparedness but also promote social inclusion (Pertiwi et al. 2019). People with disabilities can help plan for catastrophes and respond to them (Stough 2015).

Participation supports ‘Nothing About Us Without Us’, meaning that no policy is implemented without fully involving the people whose lives are affected by the decision. Meaningful participation supports empowerment of all children with special needs and marginal groups to work with organisations and institutions at the appropriate level in decision-making and determining targets and outcomes of initiatives (GFDRR 2018). Supporting this participation implies taking accountability by listening and providing feedback on stories from stakeholders and beneficiaries, which are further used to improve the service.

Children with special needs are involved in making decisions regarding DRR in schools because they are a source of information because of their condition. Therefore, they are allowed to express their opinions regarding disaster education, become facilitators of DRR in schools, and participate in training (ASB Indonesia and the Philippines 2018).

### Non-discrimination and disability-inclusive disaster risk reduction

Non-discrimination refers to equal opportunity for children with disabilities as they do not have the same starting point, compared to children without disabilities. Therefore, DiDRR must ensure that disaster risk reduction measures do not create new barriers. One important implication of non-discrimination is the systematic consideration of accessibility issues. The DiDRR implies that the physical environment, transportation, information and communication should be equally accessible to PWDs and integrated into planning and implementation processes. Stigma, prejudice and the hidden nature of some disabilities can also hinder inclusion of PWDs (King et al. 2019).

Furthermore, the school environment should be friendly and prioritise the safety of children with special needs. Access and opportunities are provided for individuals with disabilities to share their stories and experiences to eliminate social stigma. Schools ensure this through inclusive disaster risk mapping, DRR activities, humanitarian actions, and psychological support after a disaster.

### Strategy and method supporting children with special needs and disability-inclusive disaster risk reduction

Methods and strategies are essential components that support the success of learning in accordance with the needs of children with disabilities (Rofiah et al. 2021). For example, those with mild and moderate mental retardation can still receive information on rescue measures by adjusting the learning method when a disaster occurs.

Support in DiDRR is a modification of DRR learning methods for this category of children. Teachers use fun learning techniques, such as songs and concrete media with integrated materials, to teach them and regularly conduct disaster simulation exercises (Rofiah et al. 2021). Teachers create an individual education programme (IEP) for students with special needs while collaborating with the rest of the IEP team to arrange for their safety, even during a national lockdown or a natural hazard. The IEP team is ideally equipped to create and implement an individual emergency lockdown strategy because it understands such children’s intellectual, physical, emotional and health requirements (Clarke et al. 2014; Dusty, Clarke &Weber 2019). Teachers integrate disaster mitigation knowledge into teaching and learning resources (Nagata & Kimura 2020). Instead of textbooks, they relied on other sources of learning, such as local wisdom, which were used to teach many values.

### Collaboration and networking and disability-inclusive disaster risk reduction

Collaboration is needed to develop DiDRR because it is difficult to overcome these challenges individually. Furthermore, DiDRR requires people with diverse knowledge, skills and resources to work together to develop innovative solutions for people with disabilities to prepare, respond and recover during disasters (Villeneuve 2021).

Schools need to work with relevant stakeholders, such as local governments, committees, non-governmental organisations, disabled persons’ organisation, fire brigades, and the Red Cross, to complete disaster risk reduction programmes and provide options for students with various disabilities, such as those with vision or hearing problems, or using wheelchairs (ASB Indonesia and the Philippines 2018; Gartrell et al. 2020). The government should provide DRR budget support and offer regular training to increase schools’ capacities, which involve services for residents. Furthermore, parents need to be involved in the disaster socialisation and training process to support DRR activities and make the school environment safe from disasters (Kawasaki et al. 2020). Schools also need to involve non-governmental organisations, such as villages, in disaster emergency response for children with special needs (Izumi & Shaw 2012).

Based on the literature review, it can be conceptualised that the six most important aspects of DiDRR in schools are:

- the identification of students with special needs,
- accessibility,
- meaningful involvement,
- non-discrimination, strategy, and
- technique, collaboration and networking.

The reviewed literature is therefore used to create the indicator framework in this study.

## Research methods and design

This research was conducted with approval from the Medical and Health Research Ethics Committee (MHREC)Gajah Mada University, Indonesia (Protocol Number KE/0900/09/2020). The explanatory research used purposive sampling to collect data using a questionnaire survey instrument distributed to 153 principals and teachers at primary schools. The respondents were drawn from the Yogyakarta Province and included those who were familiar with the aspects of disability-inclusive DRR. The teachers and principals who worked in inclusive schools and who had previous experience with either socialisation or training pertaining to disaster education were given priority to participate in the study. Six surveys were excluded from the analysis. From these, four were incomplete, and one apiece was filled from kindergarten and high school. This left a sample of 147 completed questionnaires, which were also deemed suitable for inclusion in this study.

The DiDRR in school consists of six constructs broken down into 50 items designed to identify children with special needs (Appendix 1). These items were developed after reviewing the literature on DiDRR because there were no specific and appropriate questionnaires available to collect responses. All constructs and items were informed by relevant literature and references, focusing on factors such as the identification of children with special needs, accessibility, meaningful participation, non-discrimination, strategy and method, and collaboration and networking. The 50 items were created and measured using a five-point Likert scale, where 1, 2, 3, 4 and 5 denoted: strongly disagree, disagree, neutral, agree and strongly agree in that order. This analysis is used to obtain the loading factor estimation results from one indicator to the dimensions and latent variables. The DiDRR in School (Y) comprises several dimensions, namely identification of children with special needs (X1), accessibility (X2), meaningful participation (X3), non-discrimination (X4), and strategy and method (X5), as well as collaboration and networking (X6).

The research employed covariance-based structural equation modelling (CB-SEM) analysis with a reflective second-order confirmatory factor analysis (CFA) modelling. The study included unidirectional models from exogenous variables to constructs and indicators. The LISREL 8.80 software was utilised to examine the construct validity, reliability, and model fit in the initial and re-specification models. All data analysis procedures were conducted anonymously to ensure the confidentiality of the participants’ information.

### Ethical considerations

This research was conducted with approval from the Medical and Health Research Ethics Committee (MHREC) Gajah Mada University, Indonesia. Protocol Number KE/0900/09/2020.

## Results

The approach evaluates and assesses the ability of schools to implement DiDRR for identifying children with special needs (X1), accessibility (X2), meaningful participation (X3), non-discrimination (X4), strategy and method (X5), and collaboration and networking (X6). Disaster mitigation results in inclusive school setting assessments are used as a basis for schools to survive and systematically improve the current disaster mitigation system.

Table 1 illustrates that out of 10 goodness-of-fit (GOF) indicators, four are in a less good category or a bad fit. Meanwhile, the other indicators had marginal and good fit categories. Furthermore, the model is modified to improve its GOF because the good category is three. This initial model has no problem because all loading factor parameters have values greater than 0.5 when loading factor values are viewed. Table 2 shows that GOF value increased in Table 2 using a model specification process.

**TABLE 1:** The goodness-of-fit (GOF) index standardised model.

GOF	Acceptable match level	Model index	Explanation
Chi-square	chi-square ≤ 2 df (good fit), 2 df < chi-square ≤ 3 df (marginal fit)	2673.8	Good less
*P*-value	*P* ≥ 0.05	0.000	Good less
GFI	GFI ≥ 0.9 (good fit), 0.8 ≤ GFI ≤ 0.9 (marginal fit)	0.577	Good less
RMR	RMR ≤ 0.5	0.046	Good fit
RMSEA	0.05 < RMSEA ≤ 0.08 (good fit), 0.08 < RMSEA ≤ 1 (marginal fit)	0.094	Marginal fit
NNFI	NNFI ≥ 0.9 (good fit), 0.8 ≤ NNFI ≤ 0.9 (marginal fit)	0.973	Good fit
NFI	NFI ≥ 0.9 (good fit), 0.8 ≤ NFI ≤ 0.9 (marginal fit)	0.956	Good fit
AGFI	AGFI ≥ 0.9 (good fit), 0.8 ≤ AGFI ≤ 0.9 (marginal fit)	0.539	Good less
RFI	RFI ≥ 0.9 (good fit), 0.8 ≥ RFI ≤ 0.9 (marginal fit)	0.954	Good fit
CFI	CFI ≥ 0.9 (good fit), 0.8 ≤ CFI ≤ 0.9 (marginal fit)	0.974	Good fit

GOF, goodness of fit; GFI, goodness of fit Index; RMR, root mean square residual; RMSEA, root mean square error of approximation; NNFI, non-normed fit index; NFI, normed fit index; AGFI, adjusted goodness of fit index; RFI, relative fit index; CFI, comparative fit index.

**TABLE 2:** Goodness-of-fit index respecification model.

GOF	Acceptable match level	Model index	Explanation
Chi-square	chi-square ≤ 2 df (good fit), 2 df < chi-square ≤ 3 df (marginal fit)	1876.2	Good fit
*P*	*P* ≥ 0.05	0.000	Good less
GFI	GFI ≥ 0.9 (good fit), 0.8 ≤ GFI ≤ 0.9 (marginal fit)	0.660	Good less
RMR	RMR ≤ 0.5	0.043	Good fit
RMSEA	0.05 < RMSEA ≤ 0.08 (good fit), 0.08 < RMSEA ≤ 1 (marginal fit)	0.068	Good fit
NNFI	NNFI ≥ 0.9 (good fit), 0.8 ≤ NNFI ≤ 0.9 (marginal fit)	0.981	Good fit
NFI	NFI ≥ 0.9 (good fit), 0.8 ≤ NFI ≤ 0.9 (marginal fit)	0.966	Good fit
AGFI	AGFI ≥ 0.9 (good fit), 0.8 ≤ AGFI ≤ 0.9 (marginal fit)	0.611	Good less
RFI	RFI ≥ 0.9 (good fit), 0.8 ≥ RFI ≤ 0.9 (marginal fit)	0.963	Good fit
CFI	CFI ≥ 0.9 (good fit), 0.8 ≤ CFI ≤ 0.9 (marginal fit)	0.983	Good fit

GOF, goodness of fit; GFI, goodness of fit Index; RMR, root mean square residual; RMSEA, root mean square error of approximation; NNFI, non-normed fit index; NFI, normed fit index; AGFI, adjusted goodness of fit index; RFI, relative fit index; CFI, comparative fit index.

**FIGURE 1:**
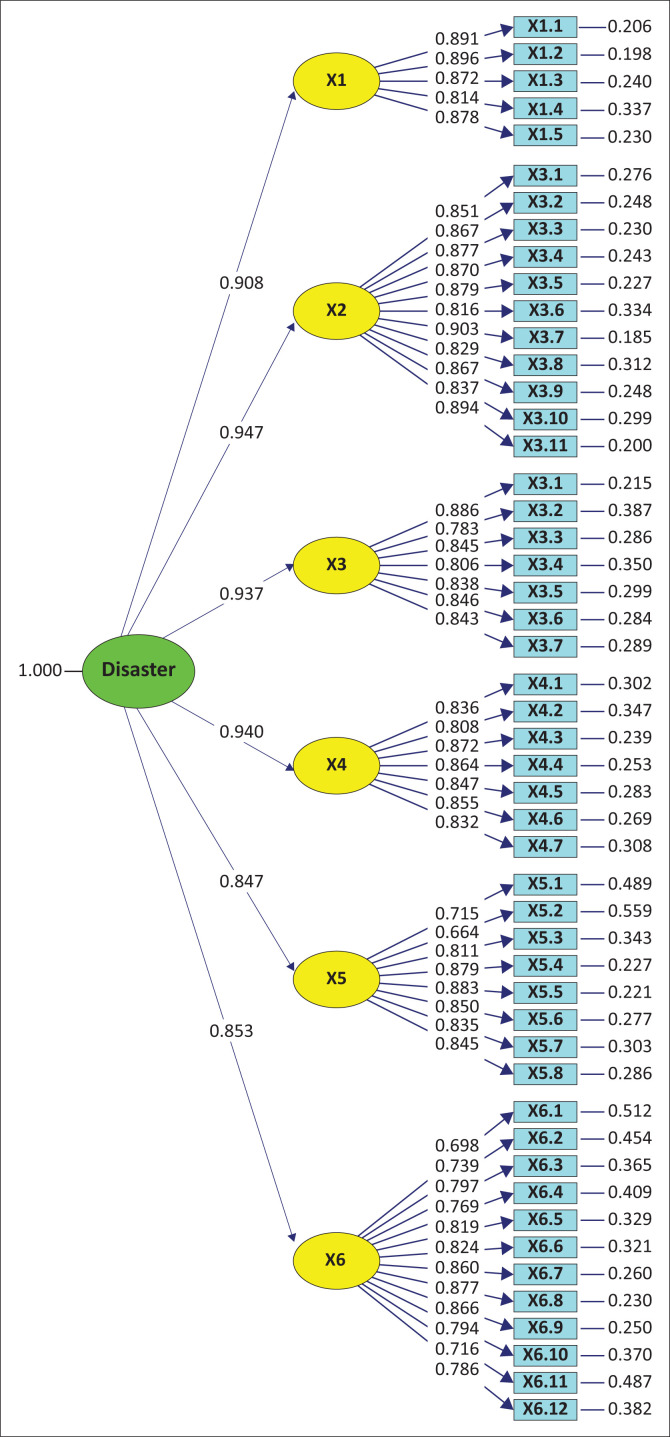
Standardised second confirmatory factor analysis model.

**FIGURE 2:**
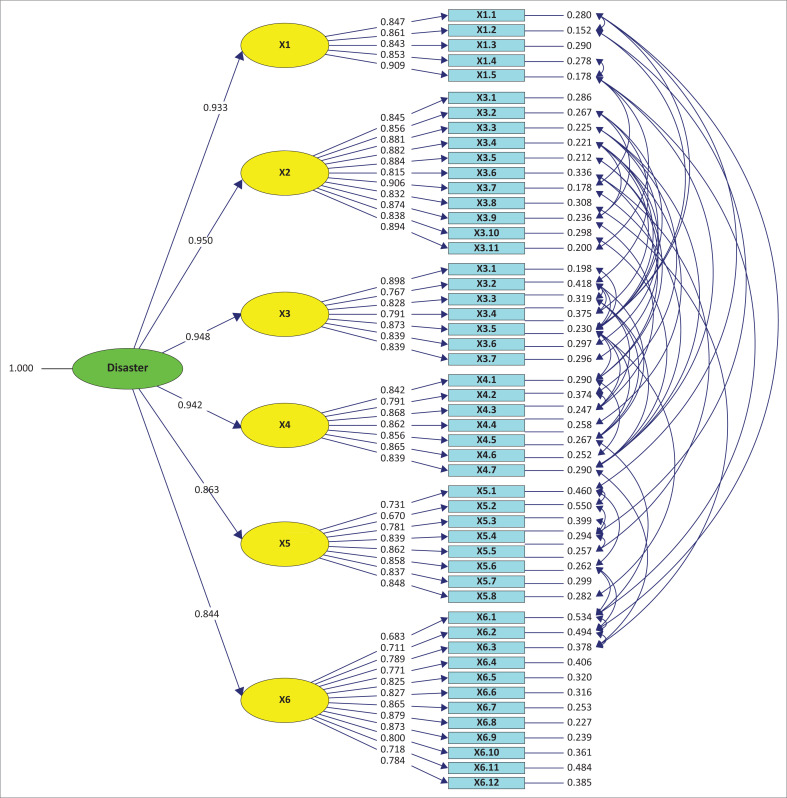
Re-specification output of second confirmatory factor analysis model.

After the model was re-specified, the GOF indicator in the less good category decreased by 3. The standardised second CFA model is shown in Figure 1 and the re-specification output of second CFA model is shown in Figure 2. Therefore, the CFA model is used to test the validity and reliability, as well as research hypothesis.

The validity and reliability analysis tests were recapitulated using the second order CFA model test. Therefore, an indicator statement is valid with a loading factor value greater than or equal to 0.05. According to Hair et al. (2010) and Ghozali and Fuad (2012), this value is reliable, assuming that the construct reliability (CR) and the variance extracted (VE) values are greater than 0.7 and 0.5. Sharma (1996) stated that the weakest acceptable factor load is 0.40. Moreover, Hair et al. (2010) stated that the construct has good reliability assuming the CR and VE values are 0.70 and 0.40, respectively. Table 3 shows the model with good validity and reliability as well as a good model fit test.

**TABLE 3:** Validity and reliability analysis results of re-specification model.

Laten variable	Manifest variable	λ	λ^2^	E	CR	VE
Identification of children with special needs (X1)	X1.1	0.847	0.717	0.283	0.936	0.745
	X1.2	0.861	0.741	0.259	-	-
	X1.3	0.842	0.709	0.291	-	-
	X1.4	0.853	0.728	0.272	-	-
	X1.5	0.910	0.828	0.172	-	-
Accessibility (X2)	X2.1	0.845	0.714	0.286	0.970	0.748
	X2.2	0.856	0.733	0.267	-	-
	X2.3	0.881	0.776	0.224	-	-
	X2.4	0.882	0.778	0.222	-	-
	X2.5	0.884	0.781	0.219	-	-
	X2.6	0.815	0.664	0.336	-	-
	X2.7	0.906	0.821	0.179	-	-
	X2.8	0.833	0.694	0.306	-	-
	X2.9	0.874	0.764	0.236	-	-
	X2.10	0.838	0.702	0.298	-	-
	X2.11	0.894	0.799	0.201	-	-
Meaningful participation (X3)	X3.1	0.898	0.806	0.194	0.941	0.697
	X3.2	0.767	0.588	0.412	-	-
	X3.3	0.828	0.686	0.314	-	-
	X3.4	0.791	0.626	0.374	-	-
	X3.5	0.873	0.762	0.238	-	-
	X3.6	0.839	0.704	0.296	-	-
	X3.7	0.839	0.704	0.296	-	-
Non-discrimination (X4)	X4.1	0.842	0.709	0.291	0.946	0.716
	X4.2	0.790	0.624	0.376	-	-
	X4.3	0.868	0.753	0.247	-	-
	X4.4	0.862	0.743	0.257	-	-
	X4.5	0.856	0.733	0.267	-	-
	X4.6	0.865	0.748	0.252	-	-
	X4.7	0.838	0.702	0.298	-	-
Strategy and method supporting children with special needs (X5)	X5.1	0.731	0.534	0.466	0.936	0.649
	X5.2	0.669	0.448	0.552	-	-
	X5.3	0.781	0.610	0.390	-	-
	X5.4	0.839	0.704	0.296	-	-
	X5.5	0.862	0.743	0.257	-	-
	X5.6	0.858	0.736	0.264	-	-
	X5.7	0.837	0.701	0.299	-	-
	X5.8	0.848	0.719	0.281	-	-
Collaboration and networking (X6)	X6.1	0.683	0.466	0.534	0.954	0.634
	X6.2	0.711	0.506	0.494	-	-
	X6.3	0.789	0.623	0.377	-	-
	X6.4	0.771	0.594	0.406	-	-
	X6.5	0.825	0.681	0.319	-	-
	X6.6	0.827	0.684	0.316	-	-
	X6.7	0.865	0.748	0.252	-	-
	X6.8	0.879	0.773	0.227	-	-
	X6.9	0.873	0.762	0.238	-	-
	X6.10	0.800	0.640	0.360	-	-
	X6.11	0.718	0.516	0.484	-	-
	X6.12	0.784	0.615	0.385	-	-

E, Error variance; CR, composite reliability; VE, variance extracted.

The results of hypothesis testing indicate that the most influential factor in DiDRR is accessibility (X2) as the estimated coefficient value for this construct was at 0.950, compared to others (Table 4). The *t*-count value is greater than 1.96, which indicates that accessibility plays a very significant role in DiDRR. The second-order of the most influential factors is meaningful participation (X3) with an estimated value of 0.948, followed by non-discrimination (X4), identification of children with special needs (X1), strategy and methods for mentoring children with special needs (X5), and collaboration and networking (X6) factors with values of 0.942, 0.933, 0.863, and 0.844, respectively. These constructs (factors) have *t*-values greater than 0.05, which means that they all have a significant effect on DiDRR.

**TABLE 4:** Hypotheses test recapitulation.

Path†	Estimate	Error variance	*R* ^2^	*t*-values ≥ 1.96	Explanation‡	Standardised loading factor ≥ 0.5	Explanation§
DiDRR → X1	0.933	0.129	0.871	11.678	Significant	0.933	Valid
DiDRR → X2	0.950	0.097	0.903	11.914	Significant	0.950	Valid
DiDRR → X3	0.948	0.100	0.900	12.902	Significant	0.948	Valid
DiDRR → X4	0.942	0.112	0.888	11.686	Significant	0.942	Valid
DiDRR → X5	0.863	0.256	0.744	8.969	Significant	0.863	Valid
DiDRR → X6	0.844	0.288	0.712	8.373	Significant	0.844	Valid

† , The direction of the causative effect;

‡ , Each indicator has a significant causative relationship with the DiDRR variable;

§ , Valid indicator in constructing DiDRR variable.

The analysed results show that the identification aspects of children with special needs, accessibility, meaningful participation, non-discrimination, strategies and methods of supporting, and collaboration and networking are positively supported by their respective behavioural indicators. Therefore, the theoretical model of DiDRR is in accordance with the empirical data. The most dominantly reflected DiDRR aspect is accessibility and the weakest aspect is collaboration and networking.

## Discussion

The data analysis indicates that the most influential factor in DiDRR from the study is accessibility. This is in line with the previous finding of Nikolaraizi et al. (2021), who found that accessibility and participation are significant challenges to implementing DiDRR in schools. This study emphasises the importance of multidisciplinary working teams and collaborative practices among schools, non-formal learning centres, and other organisations in developing accessible and inclusive DRR learning programmes for children with VI and DHH. A synthesis of theoretical ideas related to accessibility, learning, and DRR was used in the design and execution of participatory learning workshops. According to Radianti, Gjøsæter and Chen (2019), citizens need access to information about preparation and prevention of disasters. This study shows an exploratory assessment of accessibility on a collection of web pages designed to disseminate information to increase disaster risk reduction and endeavour to strengthen the community for resilience and to empower vulnerable groups and PWDs in an emergency.

The weakest aspect of DiDRR in schools is collaboration and networking. This finding echoes similar sentiments from previous findings that only a few schools collaborate with stakeholders such as education authorities, disaster management authorities, NGOs, school committees, including parents’ community, private sector, media, research centres and/or universities, and other governments (Rofiah et al. 2021). In Rofiah et al. (2021), one study found that schools did not receive any financial support from the government for DRR education; schools take independent initiatives to implement disaster risk-reduction education. The findings of Oktari et al. (2018) also mention the lack of significant progress in cooperation between schools and the community. Previous disaster education programmes did not involve local communities. However, schools have a great opportunity to collaborate with the education authority, the school committee, parents/family members, the community, and other stakeholders to enhance disaster resilience in the community.

School collaboration and networking can be a valuable and active source of support for the school while also benefiting the other stakeholders in the network. An effective collaborative network is made up of leadership, trust, facilities and infrastructure, funding resources, capacity building, awareness of all parties, regulation and an appointed team (Oktari et al. 2018). Joint activities can include developing preparedness plans together, conducting joint simulations, and raising awareness in the surrounding community with school children (Amri et al. 2017).

The principles of DiDRR include comprehensive accessibility, universal and non-discriminatory building design, coordination, and collaboration in all DRR efforts (CBM 2018; Handicap International 2014; Rothe, Brown & Neuschäfer 2018). People with disabilities and their advocacy groups have been firmly established as valid stakeholders and players in the formulation and implementation of international disaster risk reduction policies because of the Sendai Framework Disaster Risk Reduction. The use of disability-related terminology and concepts, such as accessibility, inclusion, and universal design throughout the Sendai Framework Disaster Risk Reduction text, both in relation to and apart from the mention of disability, is essential (Stough 2015).

## Conclusion

This research identified and examined the shaping factors of DiDRR in schools from the perspective of teachers and principals. In conclusion, the factors responsible for the formation of DiDRR include the identification of children with special needs (X1), accessibility (X2), meaningful participation (X3), non-discrimination (X4), and strategy and method (X5), as well as collaboration and networking (X6). Although each factor has a significant causative relationship with the DiDRR variable, accessibility is the most vital factor.

The CFA results show that the modified model fulfils the GOF criteria, leading to a DiDRR model that is fit and suitable when applied to a sample of elementary schools in Yogyakarta Province. Goodness-of-fit criteria include the root mean square error of approximation (RMSEA) value, which is less than or equal to 0.05, comparative fit index (CFI) > 0.90, and relative fit index (RFI) > 0.90. Therefore, it is considered an apt and suitable model when applied to a sample of schools. Testing of the construct in the DiDRR model is intended to give more valid information about the concepts. It also acts as a guide when implementing it in schools to reduce risk and achieve broader targets for children with special needs and disabilities.

The limitation of this study is the use of data collected from the principals and teachers at inclusive primary schools in only the Yogyakarta province of Indonesia. Future studies are suggested to further explore the application of DiDRR at various school levels in a broader area to determine the level of school safety. Future research recommends including children and parents/guardians as research participants or survey respondents.

## Appendix 1

**TABLE 1-A1:** Disability-Inclusive Disaster Risk Reduction (DiDRR) in school model identification.

Variable	Construct	Indicator	Symbol of indicator*
Disability Inclusive Disaster Risk Reduction	Identification of children with special needs	Schools identify the diversity of children with special needs. Schools identify of special tools needed by children. The school records the accessibility required data for the mobility of children with special needs. The school records the abilities and strengths of children with special needs. Information on diagnosis and treatment to complete the child’s medical history.	X1.1 X1.2 X1.3 X1.4 X1.5
	Accessibility	The school has special facilities for children with special needs. Safe classroom design and layout Class design and arrangement minimise risks Schools make learning modifications for students with special needs There are learning accommodations for children with special needs School infrastructure is tailored to the needs of the children School buildings are designed to provide accessible facilities for children Resources for learning about diversity education Schools increase the variety of learning resources and DRR facilities Support for safe infrastructure Modification of the school environment for students with special needs	X2.1 X2.2 X2.3 X2.4 X2.5 X2.6 X2.7 X2.8 X2.9 X2.10 X2.11
	Meaningful participation	Children with special needs are involved in planning disaster risk reduction. Children with special needs are involved in disaster risk assessment. Children with special needs attend disaster training at school. Children with special needs are allowed to become facilitators in DRR. Children with special needs are allowed to express their opinions. Children with special needs are a source of information on their condition. Children with special needs are involved in decision making.	X3.1 X3.2 X3.3 X3.4 X3.5 X3.6 X3.7
	Non-discrimination	Schools have rules/policies that support the involvement of children with special needs. The school environment is friendly to children. The school prioritises safety for children with special needs. Schools provide a platform and an opportunity for those most at risk to share their stories and experiences to eliminate social stigma. Schools carry out inclusive disaster risk mapping. Schools carry out DRR activities and inclusive humanitarian actions. Schools provide psychological support for children with special needs to be accessed after a disaster situation.	X4.1 X4.2 X4.3 X4.4 X4.5 X4.6 X4.7
	Strategy and Method Supporting Children with Special Needs	There is a modification in DRR learning for children with special needs. There is accommodation in DRR learning for children with special needs. The teacher uses fun learning methods, such as using songs in DRR learning. The teacher uses concrete media in DRR learning. DRR materials are integrated with learning. Teachers use peer tutor method in DRR learning. Schools conduct regular mock drills. The teacher prepares individual learning for children with special needs.	X5.1 X5.2 X5.3 X5.4 X5.5 X5.6 X5.7 X5.8
	Collaboration and networking	The residents around the school are involved in the DRR socialisation and training. The school committees are involved in disaster simulation activities. Parents are involved in disaster socialisation and training. Schools involve parents in supporting funds for DRR activities. The school works together with parents to make the school environment safe from disasters. The school committee plans DRR activities following the financial conditions of the parents. The school committee oversees the implementation of DRR. Schools involve NGOs in developing DRR materials. Schools involve village organisations in disaster emergency response. The government provides DRR budget support. The government offers regular training. Schools involve organisations that support children with special needs such as the association of parents of children with special needs.	X6.1 X6.2 X6.3 X6.4 X6.5 X6.6 X6.7 X6.8 X6.9 X6.10 X6.11 X6.12
